# The effects of a 12-week moderate-intensity continuous training intervention on depression, anxiety, and stress in sedentary female college students: a focus on negative emotion regulation

**DOI:** 10.3389/fpsyg.2025.1507198

**Published:** 2025-02-11

**Authors:** Quanwen Zeng, Yong Zhang, Huimin Li, Jin Yuan, Dan Feng, Gendi Zhu

**Affiliations:** Department of Physical Education, Anhui Polytechnic University, Wuhu, China

**Keywords:** 12 weeks, moderate-intensity continuous training, negative emotions, sedentary behavior, female college students

## Abstract

**Objective:**

Moderate Intensity Continuous Training (MICT) is recognized as an effective intervention for improving negative affect. However, research on its effects across varying levels of negative mood states in sedentary female college students remains limited. This study aimed to investigate the impact of a 12-week MICT intervention on different levels of negative mood in sedentary female college students.

**Methods:**

A total of 144 participants were randomly assigned to two groups, each consisting of 72 individuals. The participants were further categorized into three negative mood groups: depression, anxiety, and stress, with 24 participants in each group. Within each mood group, participants were divided into three subgroups based on the severity of their mood (mild, moderate, and severe), with 8 participants in each subgroup. The experiment spanned 12 weeks, with two 45-min training sessions per week. Intensity was monitored throughout the experiment using the Borg Rating of Perceived Exertion (RPE), and heart rate was measured immediately following each session. The training intensity was maintained at 60–69% of HRmax throughout the 12 weeks.

**Results:**

After 12 weeks of MICT, MICT had a positive effect on mild and severe depressive mood, moderate anxiety, and mild stressful mood in sedentary female college students (*p* < 0.05), but MICT did not have statistically significant effects on moderate depressive mood, mild and severe anxiety, and moderate and severe stressful mood in sedentary female college students (*p* > 0.05).

**Conclusion:**

MICT may have a beneficial effect on sedentary female college students, particularly those with lower levels of emotional distress. However, due to the absence of a positive control group, it is difficult to draw definitive conclusions about its specific impact. Future studies should employ more rigorous control designs to better assess the role of MICT in improving both the physical and mental health of sedentary female college students.

## 1 Introduction

Mental health is a growing concern for universities, as the transition from late adolescence to adulthood often coincides with the onset of mental health issues (Acharya et al., 2018). This period is particularly vulnerable to the development of disorders such as depression, anxiety, and substance use disorders (Blanco et al., 2008; Liu et al., 2019; Reddy et al., 2018). Research has shown that the prevalence of psychiatric conditions is notably higher among college students compared to their non-college peers and the general population (Auerbach et al., 2016; Ibrahim et al., 2013), In fact, it is estimated that nearly half of college students experience moderate stress related to mental health concerns, such as depression and anxiety (Regehr et al., 2013). The high incidence of negative emotional states among students is linked to several factors, including academic pressure, family-related stress, relationship challenges, career uncertainty, as well as unhealthy lifestyle habits, such as inadequate physical activity, poor diet, and disrupted sleep patterns (Chiang et al., 2019; Goff, 2011; Elias et al., 2011; Ketchen Lipson et al., 2015; Pedrelli et al., 2015; Kadapatti and Vijayalaxmi, 2012).

Recent research suggests that prolonged sedentary behavior may be an independent risk factor for adverse health outcomes (Keadle et al., 2017; Owen et al., 2010; Thyfault et al., 2015; Patterson et al., 2018). A significant portion of college students engage in sedentary activities (Cotten and Prapavessis, 2016), with a meta-analysis using accelerometer data showing that students spend up to 9.82 h per day in sedentary behavior (Castro et al., 2020). This trend is closely linked to lifestyle changes, particularly the rise in screen-based leisure activities (Bennie et al., 2015). Research has consistently found a negative correlation between prolonged sedentary behavior and mental health issues (Wang et al., 2019; Yue et al., 2017; Liu et al., 2016), particularly in relation to anxiety and depression. As sedentary time increases, the severity of these emotional problems also escalates (Coakley et al., 2021). Furthermore, extended periods of inactivity may contribute to the development of psychological disorders, including depression, anxiety, and bipolar disorder (Vancampfort et al., 2016; Vancampfort et al., 2017; Zhai et al., 2015).

Gender differences are evident in the prevalence of mental health issues, with research indicating that women are more susceptible to depression and anxiety disorders than men (Jinghui et al., 2020). For example, women are twice as likely as men to experience major depressive disorder (Andrade et al., 2003; Bromet et al., 2011). Studies have also found that female college students report higher levels of stress compared to their male counterparts (Evans et al., 2018; Lee et al., 2021; Moses et al., 1989; Reed et al., 2022). Furthermore, global (Hallal et al., 2012), European (Commission E, 2014), and German studies of both adult populations (Sjöström et al., 2006) and university students (Towne et al., 2017) have shown that women engage in less physical activity than men. Given the high prevalence of negative emotions and the lower participation in physical activity among female college students, it is crucial to develop effective, targeted interventions for this group.

The beneficial effects of physical activity on mental health are well established. A substantial body of evidence supports that regular physical activity can alleviate negative emotions and prevent the onset of mental illnesses (Firth et al., 2020). Research indicates that MICT is particularly effective in improving negative emotions such as depression, anxiety, and stress (Moses et al., 1989; Reed et al., 2022; Mengjiao et al., 2021; Paolucci et al., 2018). Additionally, some studies comparing the mood-modifying effects of MICT and high-intensity interval training (HIIT) have suggested that HIIT may have a more pronounced effect in certain populations (Patten et al., 2023; Borrega-Mouquinho et al., 2021). However, HIIT is not suitable for individuals with low fitness levels, including those who are sedentary or new to exercise, due to its intense nature. This can lead to low compliance and a higher risk of injury. In contrast, MICT offers a more universal approach, with moderate intensity and greater sustainability.

This study significantly advances the understanding of MICT as a psychological intervention by elucidating its differential effects on varying severities of negative affect, including mild, moderate, and severe levels of depression, anxiety, and stress. While the mental health benefits of physical activity have been well documented, our findings reveal a previously unexplored relationship: between MICT and emotional severity, and provide new insights into its mechanisms of action. By stratifying participants according to the severity of emotional distress, the present study analyzes the effectiveness of MICT in negative emotions in greater detail, rather than treating these conditions as homogeneous.

## 2 Materials and methods

### 2.1 Participants

Participants were recruited through posters, flyers, and a mind–body conditioning boot camp at a local university campus. Initially, 162 individuals were recruited, and 144 were retained, divided into an experimental group (MICT) and a control group (CON), with 72 participants in each group. The mean age of the sedentary female students with depression, anxiety, and stress in this study was 18.53 ± 0.50 years, with a mean height of 166.86 ± 7.83 cm, mean weight of 57.39 ± 9.83 kg, mean BMI of 20.43 ± 2.60 kg/m^2^, and average sedentary time of 8.44 ± 2.44 h per day.

Inclusion criteria: (1) female college students aged 18–19 years and able-bodied, (2) no regular physical activity according to international standards (screened using the short version of the Physical Activity Questionnaire SF-IPAQ), (3) a score of ≥160 on the Symptom Self-Criticism Scale (SCL-90), (4) at least 6 h of sedentary behavior per day, (5) no history of psychotropic drug use, (6) no history of chronic disease, and (7) attendance at all training sessions during the intervention cycle. Exclusion criteria: (1) inability to perform physical activity at normal intensity, and (2) unexcused absences from one-third or more of the experimental sessions.

The study adhered to the Declaration of Helsinki and was approved by the Ethics Committee of the Institute of Neuroscience and Cognitive Psychology at the local university. Participants were fully informed about the study’s purpose, procedures, and potential risks, and provided informed consent. Confidentiality of personal information was guaranteed to protect participants’ privacy. Throughout the intervention cycle, no participants withdrew, all training sessions were completed, and no adverse effects were reported (Table 1).

**Table 1:** Baseline characteristics of participants.

Characteristic	Participants-MICT	Participants-CON	All
*n*	72	72	144
Age (years)	18.63 ± 0.49	18.43 ± 0.50	18.53 ± 0.50
Height (cm)	166.94 ± 8.18	166.78 ± 7.53	166.86 ± 7.83
Weight (kg)	56.83 ± 9.01	57.94 ± 10.65	57.39 ± 9.83
BMI (kg/m^2^)	20.42 ± 2.69	20.44 ± 2.54	20.43 ± 2.60
SE	8.46 ± 2.36	8.41 ± 2.54	8.44 ± 2.44
Scl-90	≥160	≥160	≥160

BMI, body mass index; SE, sedentary time; SCL-90, Symptom Self-Rating Scale.

### 2.2 Research design and procedures

**Figure 1:**
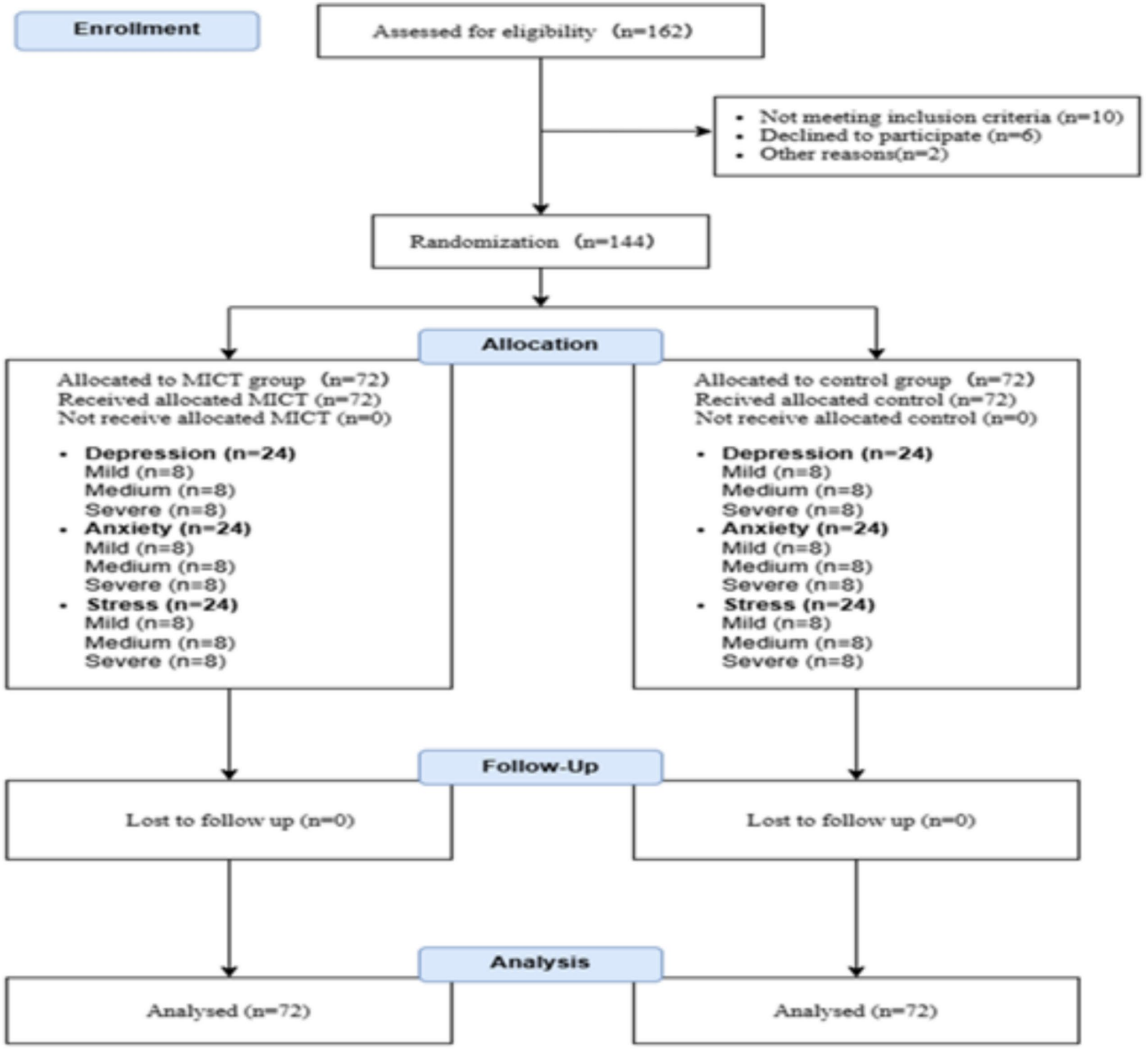
Consolidated Standards of Reporting Trials (CONSORT) diagram showing the flow diagram of participants.

In this study, a preliminary screening of the target group, female college students, was conducted through a questionnaire survey. The questionnaire included basic socio-demographic information such as name, age, profession, and contact details, alongside the Simplified International Physical Activity Questionnaire (SF-IPAQ), a reliable tool for assessing sedentary behavior, and the Symptom Self-Control Scale (SCL-90). The following steps were undertaken to complete the screening process (as outlined in the CONSORT flow diagram, Figure 1):

(1) Initial Screening: Researchers collected data on participants’ emotional states and physical activity levels over a one-week period using the SF-IPAQ and SCL-90. Participants with SCL-90 scores of 160 or above were selected (A score of 160 or higher on the SCL-90 indicates a significant level of psychological distress, suggesting possible mental health issues or mood disorders). Additionally, participants who were sedentary for at least 6 h per day were included, ensuring that the subjects exhibited both elevated emotional distress and high levels of sedentary behavior, aligning with the study’s objectives.

(2) Further Screening: One week before the official start of the experiment, the height and body composition of the screened participants were measured, and they were asked to complete the Depression Anxiety Stress Scale (DASS-21). This scale, known for its good reliability and validity, provides an accurate assessment of depression, anxiety, and stress levels (Xu et al., 2010). The DASS-21 is composed of three dimensions—depression, anxiety, and stress—each with seven items, totaling 21 questions. The severity of each item is rated on a scale from 0 to 3, with scores for each dimension calculated by summing the responses and multiplying by two (The scoring criteria for each dimension are as follows: Depression: A score of ≤9 is considered normal, 10–13 indicates mild depression, 14–20 is moderate, 21–27 is severe, and a score of ≥28 is extremely severe. Anxiety: A score of ≤7 is normal, 8–9 is mild, 10–14 is moderate, 15–19 is severe, and ≥20 is extremely severe. Stress: A score of ≤14 is normal, 15–18 is mild, 19–25 is moderate, 26–33 is severe, and ≥ 34 is extremely severe). These criteria were used to assess participants’ emotional states and finalize the selection of experimental subjects.

(3) Experimental Staffing and Roles: To ensure the smooth execution of the experiment and the accuracy of data, five assistant researchers and three volunteers were recruited. The assistant researchers, all master’s degree students in physical education, were primarily responsible for helping participants complete the questionnaires, recording physiological data, and overseeing the experimental process. The volunteers supported the assistant researchers by conducting heart rate tests and administering the Borg Rating of Perceived Exertion (RPE) (Ritchie, 2012) scale at the beginning, during, and after the experiment.

(4) Conduct of the Experiment: All tests were conducted between September and December at a local university physical fitness center. To control for potential confounding factors, participants were asked to maintain their usual diet, study, and exercise routines throughout the experiment. No additional exercise interventions were permitted, aside from the intervention outlined in the study.

### 2.3 Interventions

Martland et al.’s study shows that the duration of MICT intervention needs to be ≥2 weeks, and the frequency is twice a week (Martland et al., 2022). Considering the physical and psychological characteristics of female college students who have been sedentary for a prolonged period, lack regular exercise, and experience negative emotions, excessive intensity or frequency may lead to reduced interest and lower participation rates. This, in turn, could hinder the completion of the intervention as intended. Therefore, this study follows the approach suggested by Reed (Reed et al., 2022) and other scholars, with the MICT intervention set at two sessions per week for 12 weeks, totaling 24 sessions.

The experimental group’s training program consisted of two 45-min MICT sessions per week. Each session began with a 10-min warm-up, followed by 30 min of MICT, and concluded with 5 min of static stretching. The MICT portion of the program involved four exercises, each lasting 1 min with no breaks in between. These exercises were repeated twice for a total of three sets, with a 2-min rest between each set. The control group, maintained their usual study and exercise habits without any additional intervention.

For the intervention, we selected exercise combinations that were both engaging and highly stimulating, considering the participants’ long-term sedentary behavior and emotional challenges (see Table 2 for details). The 30-min moderate-intensity continuous training (MICT) required participants to perform each exercise for 60 s without any breaks. Each training session consisted of two rounds, each lasting 8 min, with a 2-min rest between rounds. During the rest period, participants were instructed to remain seated and refrain from talking or engaging in any physical activities with other participants or staff.

**Table 2:** The organization of the content of MICT.

Phase	Week	Training content	Time	Exercise loads
Foundation stage	1–6 week	Open and close jumps Squats Fast trot in place 10 m fast back and forth run	4x60sx2 (Four moves) 3X4x60sx2 (Four moves × three sets) Rest time 2 min (no exercise)	60–69%HRmax
Advancement stage	7–12 week	Bobbi Jumps Abdominal jumps In-situ high kicks 10 m fast back and forth run		

### 2.4 Measurement

#### 2.4.1 Sedentary behavior

To ensure the accuracy of the assessment of sedentary behavior, this study requested that all subjects complete the SF-IPAQ questionnaire before each week’s formal training, with a view to recording their daily sedentary time over the preceding 7 days. Subjects whose sedentary time did not meet the criteria (≥6 h/day) or whose sedentary behavior was significantly reduced due to lifestyle changes were excluded from the study based on the data obtained from the questionnaire.

#### 2.4.2 Heart rate

Immediately following the conclusion of each training session, the research assistant provided the subjects with instructions to measure their heart rates. The measurement was conducted by placing the index and middle fingers of the right hand on the radial artery of the left hand, recording the number of pulse beats in 15 s by palpation, and multiplying this value by four to calculate the one-minute heart rate (HR). The training heart rate will be compared to the subject’s maximum heart rate (HRmax), calculated using the formula HRmax = 207–0.7 × age, as proposed by Gelish, to ascertain whether the exercise intensity is aligned with the prescribed target (60–69% HRmax).

#### 2.4.3 Consciousness of fatigue

Prior to the commencement of the experiment, participants were provided with instructions on how to complete the Borg RPE scale, thus ensuring a consistent approach to data collection. During the basic training, a questionnaire was administered by the researchers and auxiliary personnel at designated rest periods between each group of exercises. The subjects were asked to indicate the subjective intensity of the exercise they had just completed, and the time required to complete the scale was 15–30 s. The scale was used to evaluate the extent of subjective fatigue experienced during individual exercises. The scale employs a 6–20 scoring range, with 6–11 indicating no effort and relative easy, 12–14 indicating somewhat strenuous, 15–18 indicating strenuous and very strenuous, 19 indicating extremely strenuous, and 20 indicating complete exhaustion.

#### 2.4.4 Depression-anxiety-stress mood level (DASS-21)

To evaluate the psychological state of participants following the training program, the DASS-21 scale was employed. The scale is a simplified version of a mental health assessment tool comprising 21 entries for the assessment of depression, anxiety, and stressful emotions, with seven entries for each dimension. The scores are categorized into five levels: normal, mild, moderate, severe, and very severe, according to a standardized scoring guide, which allows for the differentiation of the severity of emotional problems. DASS-21 has been demonstrated to have good cross-cultural applicability, psychometric reliability, and validity.

### 2.5 Statistical analysis

The sample size was selected using the G-Power software and a two-tailed t-test (means). A two-tailed, two-sample t-test was used to assess the difference between two independent means (two groups), with a medium effect size (*d* = 0.5, *α* = 0.05, statistical power = 0.8) deemed necessary for statistical significance. This resulted in a total sample size of 128, with 64 participants in each group. Considering the potential for a natural attrition rate during the experiment, 162 participants were initially recruited, with 144 ultimately included in the final experimental analysis, comprising 72 participants in each group. Participants were divided into three categories according to their mood states, with 24 individuals assigned to each of the depression, anxiety, and stress groups. Within each group, three severity levels were considered, with eight individuals each in the mild, moderate, and severe categories.

Data were analyzed using IBM SPSS Statistics version 27. The normality of the data was evaluated using the Shapiro–Wilk test, which substantiates the assumption that the data are normally distributed, thereby enabling the use of parametric tests. The level of significance was set at *p* < 0.05. First, within-group comparisons were conducted using paired-samples *t*-tests for the experimental group (MICT) and control group (CON). This was done to assess the change in mood scores within each group from the preexperiment to the end of the experiment. This analysis assessed the effectiveness of the intervention on varying degrees of negative moods in each group. Subsequently, between-group comparisons were conducted using independent sample t-tests to compare post-experiment outcome scores between the experimental and control groups. Given the limited sample size of each group (*n* < 30), Hedges’ *g* was deemed an appropriate reference indicator for effect size, small effect (0.2 ≤ *g* < 0.5); medium effect (0.5 ≤ *g* < 0.8); large effect (*g* ≥ 0.8).

Given that multiple comparisons were made, In this study, we used the Benjamini-Hochberg (BH) method to correct *p*-values for multiple hypothesis tests to control for false discovery rate (FDR). The method arranges the p-values in order from smallest to largest and calculates the critical point for each *p*-value according to the following equation $BH_{\mathrm{critical}}=\frac{i\cdot \alpha }{N}$. Here, 𝑖 represents the rank of the *p*-value, 𝑁 is the total number of tests, and 𝛼 is the significance threshold, set at 0.05 in this study. A test was deemed statistically significant if its p-value was less than or equal to its corresponding critical value. This approach strikes a balance between minimizing false positives and maintaining statistical power. Unlike overly conservative methods, the BH adjustment avoids unnecessarily reducing the likelihood of detecting true effects. Any corrected p-values exceeding 1 were capped at 1 and considered insignificant.

## 3 Results

### 3.1 Comparison within groups

#### 3.1.1 Experimental group

Depression: After 12 weeks of MICT intervention, we found that the exercise had a significant effect on depressed mood. Specifically, after correcting the p-values by the BH correction method, the results showed that MICT had a significant effect on both mild and severe depressive mood (corrected p-values of 0.025 and 0.006, respectively), but did not reach the level of significance on moderate depressive mood.

Anxiety: After 12 weeks of MICT intervention, corrected for BH, we found that MICT had a significant effect on moderate anxiety mood (corrected *p*-value of 0.017). However, the intervention effect of MICT did not reach the level of significance for mild and severe anxiety mood.

Stress: 12-week MICT intervention also showed some effect in stressful mood. By analyzing the BH-corrected p-value, we found that MICT had a significant effect on mild stressful emotions (corrected *p*-value of 0.006). However, for moderate and severe stressful emotions, the MICT intervention did not show significant improvement.

Overall, the 12-week MICT intervention positively affected the regulation of mild and severe depressive moods, moderate anxiety, and mild stress in sedentary female college students.

#### 3.1.2 Control group

Under the no-exercise intervention, the control group showed no significant changes in the indicators of depression, anxiety, and stressful emotions (*p* > 0.05), indicating that the 12-week no-exercise intervention failed to improve the negative emotional states of sedentary female college students.

### 3.2 Comparison between groups

After 12 weeks of MICT, the experimental group showed significantly greater improvements in severe depressive mood and mild stressful mood compared to the control group. These results suggest that the MICT intervention has a positive effect on alleviating severe depressive mood. However, no significant differences were observed between the groups for mild and moderate depression, anxiety across all levels, or moderate and severe stressful mood. These findings highlight the specific impact of MICT on certain emotional states while indicating its limited effect on others.

In conclusion, the 12-week MICT intervention significantly improved mild and severe depressive moods, moderate anxiety, and mild stress in sedentary female college students. In between-group comparisons, the MICT intervention showed a significant advantage over the no-exercise control group in reducing severe depressive moods and mild stress. However, there were no statistically significant differences between the groups for any level of anxiety or for mild and moderate depressive and stress moods (Tables 3–5).

**Table 3:** Differences in pre-and post-test results within the experimental group.

Type (MICT)	Degree	Pre	Post	*t*	Hedges’*g*	*p* (Unadjusted)	*p* (Adjusted)	Significant
Dep	Mild	10.25 ± 0.71	9.00 ± 1.07	3.41	1.142	0.011	0.025	True
	Mod	17.50 ± 1.41	15.50 ± 2.07	2.37	0.791	0.050	0.028	False
	Sev	23.50 ± 1.77	16.25 ± 3.45	5.13	1.717	0.001	0.006	True
Anx	Mild	8.00 ± 0.00	7.00 ± 1.51	1.87	0.625	0.104	0.039	False
	Mod	12.75 ± 1.49	11.00 ± 1.07	3.86	1.291	0.006	0.017	True
	Sev	18.50 ± 2.98	17.75 ± 3.45	0.81	0.272	0.442	0.050	False
Str	Mild	16.75 ± 1.04	12.25 ± 1.28	7.18	2.399	<0.001	0.006	True
	Mod	21.00 ± 1.51	17.25 ± 4.40	2.10	0.700	0.074	0.033	False
	Sev	27.50 ± 1.41	24.25 ± 6.27	1.60	0.534	0.154	0.044	False

**Table 4:** Differences in pre-and post-test results of the control group.

Type (CON)	Degree	Pre	Post	*t*	Hedges’*g*	*p* (Unadjusted)	*p* (Adjusted))	Significant
Dep	Mild	11.00 ± 1.07	11.00 ± 1.85	0.00	0.000	1.000	0.033	False
	Mod	16.50 ± 2.07	16.75 ± 2.82	−0.357	−0.119	0.732	0.028	False
	Sev	23.50 ± 1.77	23.25 ± 1.49	1.00	0.334	0.351	0.006	False
Anx	Mild	8.00 ± 0.00	8.00 ± 1.07	0.00	0.000	1.000	0.033	False
	Mod	11.00 ± 1.51	11.00 ± 1.07	0.00	0.000	1.000	0.033	False
	Sev	16.75 ± 1.04	17.00 ± 1.07	−1.00	−0.334	0.351	0.006	False
Str	Mild	16.75 ± 1.04	16.50 ± 1.41	0.424	0.142	0.685	0.022	False
	Mod	21.50 ± 1.41	21.50 ± 0.93	0.00	0.000	1.000	0.033	False
	Sev	27.25 ± 3.54	26.75 ± 2.82	1.00	0.334	0.351	0.006	False

**Table 5:** Results of intergroup differences between the experimental and control groups after the intervention.

Type and Degree	M&C	Mean	*t*	Hedges’*g*	*p* (Unadjusted)	*p* (Adjusted)	Significant
MILD-DEP	MICT	9.00 ± 1.07	−2.642	−1.250	0.019	0.017	False
	CON	11.00 ± 1.85					
MOD-DEP	MICT	15.50 ± 2.07	−1.012	−0.478	0.329	0.039	False
	CON	16.75 ± 2.82					
SEV-DEP	MICT	16.25 ± 3.45	−5.265	−2.488	<0.001	0.006	True
	CON	23.25 ± 1.49					
MILD-ANX	MICT	7.00 ± 1.51	−1.528	−0.722	0.149	0.028	False
	CON	8.00 ± 1.07					
MOD-ANX	MICT	11.00 ± 1.07	0.000	0.000	1.000	0.050	False
	CON	11.00 ± 1.07					
SEV-ANX	MICT	17.75 ± 3.45	0.587	0.277	0.573	0.044	False
	CON	17.00 ± 1.07					
MILD-STR	MICT	12.25 ± 1.28	−6.298	−2.977	<0.001	0.006	True
	CON	16.50 ± 1.41					
MOD-STR	MICT	17.25 ± 4.40	−2.674	−1.264	0.029	0.022	False
	CON	21.50 ± 0.93					
SEV-STR	MICT	24.25 ± 6.27	1.028	−0.486	0.321	0.033	False
	CON	26.75 ± 2.82					

Dep, depression; Anx, anxiety; Str, stress; MOD, moderate; SEV, severe; con, control subjects.

## 4 Discussion

This study found that 12 weeks of MICT effectively improved mild and severe depressive moods, moderate anxiety, and mild stress in sedentary female college students. In contrast, the control group, with no exercise intervention, showed no significant improvement in any level of depression, anxiety, or stress. Between-group comparisons revealed that MICT was significantly more effective than control in reducing severe depressive mood and mild stress. However, no statistically significant differences were found between the groups for any level of anxiety or for mild and moderate depressive and stress moods. These findings offer preliminary evidence supporting the use of MICT interventions to enhance mental health, although the selective effects on different mood types and levels warrant further investigation.

### 4.1 Differential effects of MICT on different levels of depression

The 12-week MICT intervention significantly improved mild and severe depressive moods in sedentary female college students but did not have a statistically significant effect on moderate depressive mood. This differential effect may be related to psychological and physiological characteristics associated with varying levels of depression.

In case of mild depression, which are primarily characterized by low mood and cognitive symptoms (Howland et al., 2008). In these cases, neurotransmitter function has not yet been severely impaired, and exercise may alleviate symptoms by boosting the levels of dopamine, *β*-endorphin, and serotonin (Schuch et al., 2011). These neurotransmitters are vital to mood regulation and mental health. Increased levels of dopamine and serotonin play a key role in improving mood and reducing depression (Hossain et al., 2024; Martinsen, 1990; Dinas et al., 2011). Additionally, endorphins released during physical activity (Bruce, 2022) contribute to a sense of euphoria and well-being (Ali et al., 2021), which may partially explain the positive outcomes observed in this study.

Our research findings on severe depression are consistent with prior studies by Brush et al. (2022), Olson et al. (2017), and Schuch et al. (2017), showing that exercise can markedly alleviate symptoms of severe depression. Severe depressive symptoms are closely linked to systemic inflammation, as evidenced by elevated levels of pro-inflammatory cytokines such as interleukin-6 and interleukin-1β (Miller and Raison, 2016).

Compared to healthy individuals, those with severe depression exhibit elevated baseline levels of these inflammatory markers (Kruse et al., 2018). Exercise has demonstrated efficacy in ameliorating severe depression at the physiological level by stimulating the release of brain-derived neurotrophic factor (BDNF), a crucial protein that enhances neuroplasticity and supports the repair and growth of neural pathways, while also modulating inflammatory responses. Furthermore, a sedentary lifestyle has been identified as a modifiable risk factor for severe depression, underscoring the significance of exercise as an intervention. Olson et al. (2017) found that 8 weeks of moderate-intensity aerobic exercise significantly reduced depression symptoms in patients with major depressive disorder. In addition to exercise, current research explores other interventions, such as electrical stimulation, to alleviate moderate depressive symptoms (Kruse et al., 2018; Wang et al., 2024).

Our study found that 12 weeks of MICT did not significantly reduce moderate depressive moods in sedentary female college students. This may be due to the complex pathophysiology of moderate depression, which lies between the mild and severe forms. MICT alone may not be sufficient to address neurotransmitter imbalances and inflammatory responses characteristic of moderate depression, leading to a diminished overall effect in this group. Therefore, more targeted interventions are necessary to specifically address neuroinflammation and promote neuroplasticity in individuals with moderate depression.

### 4.2 The selective effect of MICT on anxiety

Our study revealed a significant improvement in moderate anxiety after 12 weeks of MICT in sedentary female college students, whereas no statistically significant changes were observed in mild or severe anxiety. This selective effect may be attributed to the distinct physiological and psychological responses of moderately anxious individuals, which may help regulate neurotransmitter activity and lead to improvements in anxiety levels. Previous studies have shown that physical activity enhances mood and improves both performance and psychological well-being in healthy individuals, including athletes (Matta Mello Portugal et al., 2013). This positive effect is partly attributed to the ability of exercise to stimulate the release of neurotransmitters such as dopamine, serotonin and endorphins. These neurotransmitters play a crucial role in mood regulation, and their increased levels can enhance individual pleasure and positively affect mood.

Several potential pathways may be involved in the mechanisms through which MICT alleviates moderate depressive moods. First, MICT may indirectly affect tryptophan utilization by promoting changes in body function and metabolism, which in turn may positively affect serotonin synthesis. Tryptophan and serotonin plays an important role in mood regulation (Smith et al., 1997), and an increase in serotonin may be beneficial for alleviating individuals with moderate anxiety (Moncrieff et al., 2023). Second, physical activity has anti-inflammatory effects (Pranoto et al., 2023), which reduces the inflammatory response of the body by reducing pro-inflammatory cytokines (e.g., interleukin-6 and tumor necrosis factor-*α*) and promoting the release of anti-inflammatory substances, which can also alleviate the symptoms of moderate anxiety to some extent (Blackman et al., 2024). Furthermore, MICT can increase the level of BDNF (Maroofi et al., 2022), promotes neurogenesis and improves synaptic connectivity, which can help alleviate anxiety symptoms (Jemni et al., 2023).

However, it is noteworthy that our study showed no significant improvement in mild or severe anxiety levels after 12 weeks of MICT. For individuals with mild anxiety, it may be that their neurotransmitter levels, inflammatory response or neuroplasticity are not significantly imbalanced. Therefore, 12 weeks of MICT did not induce significant changes in mood state. For those with severe anxiety, more complex underlying neural mechanisms might exist and require targeted interventions to improve emotional states substantially.

Although some previous studies have reported positive effects of exercise on alleviating anxiety symptoms, these effects are often mild and vary across studies (Stubbs et al., 2017). For example, some studies have found that MICT can improve anxiety in both adolescents and middle-aged to older adults (Mcmahon et al., 2017; Herring et al., 2024), but some studies have reported contradictory results. For example, Viana (Viana et al., 2019) conducted an eight-week study with two different exercise modalities and found no improvement in anxiety symptoms among healthy women. A meta-analysis by Bartley (Bartley et al., 2013) oncluded that aerobic exercise does not significantly reduce anxiety. These differences may be due to various factors such as study design, sample selection, intensity of exercise, and duration.

The differences between our findings and those of previous studies may be attributed to various factors, including the type of exercise intervention, participant characteristics, and classification of anxiety by severity levels. While some studies suggest that MICT can effectively reduce anxiety when performed for a moderate duration (Stubbs et al., 2017; Gordon et al., 2017; Cliff et al., 2016), current studies increasingly focus on the psychological and physiological mechanisms underlying anxiety reduction through exercise. These mechanisms involve factors such as cortisol reduction, neuroplasticity, and changes in inflammatory cytokine levels. Our study further supports the potential of MICT as an effective intervention to alleviate moderate anxiety.

### 4.3 MICT specific regulation of stressful emotions

In terms of improving stressful moods, our 12-week MICT was found to reduce mild stress in sedentary female college students but had no significant effect on those experiencing moderate or severe stress. This suggests that MICT may be particularly effective in reducing mild stress, but that its benefits are limited to patients with chronic or severe stress. The differential effects of MICT can be attributed to a variety of physiological and psychological factors, including the complex interplay of stress hormones, neurotransmitter regulation, and neuroplasticity.

MICT reduces stress by modulating stress hormones and promoting neuroplasticity. Studies have shown that regular MICT helps to regulate cortisol levels (the main hormone released by the body in response to stress) (Sharifabadi et al., 2022), leading to better regulation of stress and a reduction in the body’s physiological response to external stress (Mücke et al., 2018). This effect was particularly evident in mildly stressed emotional individuals, which is consistent with the results of our study. In addition to hormonal modulation, MICT promotes neuroplasticity by increasing the expression of BDNF, which improves cognitive function and enhances an individual’s ability to adapt to stress (Pranoto et al., 2020) and BDNF is critical for cognitive function and emotional resilience. Higher levels of BDNF during exercise are positively correlated with an individual’s improved ability to cope with stress (Russell et al., 2014). This mechanism explains why MICT is effective in relieving mild stress by enhancing central nervous system (CNS) function (Nowacka-Chmielewska et al., 2022). However, chronic stress often leads to deeper neurocircuitry changes and increased neuroinflammation, which MICT alone may not be sufficient to address (Hassamal, 2023; Chesnokova et al., 2016). Chronic stress is often accompanied by high levels of neuroinflammation, a state closely associated with mood disorders such as anxiety, depression, and elevated stress levels (Hassamal, 2023). While exercise, including MICT, has been shown to reduce neuroinflammation and attenuate stress-induced brain damage, it may take more time for these benefits to become apparent. For example, a related study showed that stress levels were not significantly reduced after 4 weeks of MICT, while a significant decrease was observed after 8 weeks of training (Yifan, 2021). This delayed effect emphasizes the gradual nature of the psychological and physiological adaptation processes involved in stress regulation.

Twelve weeks of MICT training had no significant effect on moderate and severe stress levels in sedentary female college students, which may be due to the mandatory exercise design of the study. The mandatory nature of the exercise itself may also have been a source of stress for participants (Lichtenstein et al., 2017). For individuals with high initial stress, the mandatory nature of the program may exacerbate rather than alleviate stress. Additionally, individual differences in stress sensitivity could explain why MICT had a more noticeable effect on mild stress than on moderate or severe stress. Individuals with higher baseline stress levels may be more reactive to external stressors, making it challenging for MICT to yield significant results. In such cases, a more holistic approach that combines exercise with other stress management techniques, such as positive thinking or cognitive-behavioral strategies, may be more effective.

### 4.4 Ineffectiveness of the control group validates the positive effects of MICT

The results of the control group demonstrated no significant changes in depression, anxiety, or stress among sedentary female college students who did not participate in the exercise intervention. This further highlights the unique effectiveness of MICT in improving mild and severe depressive mood, moderate anxiety, and mild stress. The inclusion of the control group data strengthened the comparison, making the between-group differences even more pronounced and underscoring the intervention’s positive impact.

While previous studies have noted that MICT can be monotonous, lengthy, and difficult for participants to sustain over time, our 12-week study observed full adherence from all 144 participants, with no dropouts, injuries, or adverse events. This may be attributed to the varied and progressive exercise regimen we implemented, which likely helped maintain participant engagement and minimize risk.

### 4.5 Strengths and clinical significance

Our findings examined varying responses to MICT across different emotional states, This helps us to understand the moderating effect of exercise on different levels of negative emotions. Traditional mental health interventions, such as therapy and medication, often require substantial resources and have limitations in accessibility. In contrast, MICT offers a low-cost, efficient alternative that not only supports mental health but also improves physical fitness and overall well-being.

Throughout the experiment, emotional responses were influenced by peer interactions. Some students, initially fatigued, were motivated to continue due to the positive influence of others. Participants also became more proactive in managing negative emotions during the study. Notably, many students were reluctant to seek traditional counseling services, especially those with moderate emotional distress. However, these same students showed a greater willingness to engage in group exercise, suggesting that MICT could be an accessible alternative for those avoiding conventional mental health support.

Our study was not intended to exaggerate the utility or cost-effectiveness of MICT or diminish the value of other exercise or non-exercise interventions in promoting students’ physical and mental health. While MICT improved depression, anxiety, and stress levels (mild and severe depression, moderate anxiety, and mild stress) in sedentary female college students in this study, we did not include an alternative exercise intervention or active control group for comparison. Therefore, we cannot assess the relative efficacy of MICT compared to other treatment options or its cost-effectiveness. The primary aim was to demonstrate MICT’s potential as an alternative for addressing psychological challenges in sedentary students. Future research should examine MICT’s comparative advantages through controlled studies and explore its integration into existing wellness programs.

### 4.6 Limitations and future directions

This study has several limitations that should be addressed in future research. (1) The lack of a positive control group limits conclusions about MICT’s relative effectiveness. Future studies should include a positive control, such as MBI, medication, or alternative exercise modalities, for clearer comparisons. (2) The absence of long-term follow-up data is another limitation. As previous studies show sustained benefits of exercise on mood, future research should include follow-up periods at 6 and 12 months to assess the persistence of MICT’s effects. (3) The sample was limited to female college students from a single university, restricting generalizability. Including diverse samples will enhance external validity. (4) The study only analyzed overall sedentary behavior, not its specific impacts on mood. Future research could explore how varying levels of sedentary time influence emotional health across different groups. (5) Finally, this study focused only on MICT, omitting other exercise modalities. Comparing MICT with alternatives such as HIIT or strength training will provide a broader understanding of exercise interventions and their impact on mood.

## 5 Conclusion

This study found that 12 weeks of MICT significantly improved mild and severe depressive moods, moderate anxiety, and mild stress in sedentary female college students. These findings suggest that MICT may serve as an effective, low-cost intervention to enhance both physical and mental health in this population. However, more intensive interventions may be necessary for individuals with more severe symptoms. Future research should focus on longer follow-up periods and explore multimodal interventions to further assess the potential of MICT to promote mental health.

## Data Availability

The original contributions presented in the study are included in the article/supplementary material, further inquiries can be directed to the corresponding author/s.
